# Supplementary material for: Polyketide synthase gene clusters in the ericoid mycorrhizal fungus Oidiodendron maius: genome-wide prediction, expression profiling, and functional characterization of knockout mutants
Source: Front Plant Sci. 2026 Sep 16;17:1924430. doi: 10.3389/fpls.2026.1924430 (PMC13623903; doi:10.3389/fpls.2026.1924430)
Supplement: Supplementary file 1 [file SupplementaryFile1.zip › Supplementary Materials/Supplementary Folder 1.zip!/Oidma1_AssemblyScaffolds_Repeatmasked/knownclusterblast/cluster32/jgi.p_Oidma1_130385_mibig_hits.html]

| MIBiG Protein | Description | MIBiG Cluster | MiBiG Product | % ID | % Coverage | BLAST Score | E-value |
| --- | --- | --- | --- | --- | --- | --- | --- |
| AGO86666.1 | putative\_MFS\_transporter | BGC0001255 | Equisetin\_biosynthetic\_gene\_cluster | 49 | 96.3488843813 | 443 | 4.2e-124 |
| AGO65989.1 | putative\_MFS\_transporter | BGC0000992 | Fusaridione\_A\_biosynthetic\_gene\_cluster | 49 | 97.1602434077 | 438 | 1.4e-122 |
| ACD39772.1 | major\_facilitator\_superfamily\_transporter | BGC0000134 | Radicicol\_biosynthetic\_gene\_cluster | 44 | 98.5801217039 | 407 | 2e-113 |
| KFH48645.1 | putative\_transporter\_-like\_protein | BGC0000317 | Cephalosporin\_biosynthetic\_gene\_cluster | 47 | 95.7403651116 | 395 | 7.7e-110 |
| BAK26557.1 | putative\_MFS\_multidrug\_transporter | BGC0000977 | Cyclopiazonic\_acid\_biosynthetic\_gene\_cluster | 43 | 93.509127789 | 389 | 4.2e-108 |
| ADI24938.1 | VrtL | BGC0000168 | Viridicatumtoxin\_biosynthetic\_gene\_cluster | 37 | 94.1176470588 | 291 | 1.6e-78 |
| KFH48706.1 | putative\_MFS-type\_transporter-like\_protein | BGC0000317 | Cephalosporin\_biosynthetic\_gene\_cluster | 36 | 93.509127789 | 275 | 8.9e-74 |
| EAU32817.1 | conserved\_hypothetical\_protein | BGC0000160 | Terreic\_acid\_biosynthetic\_gene\_cluster | 36 | 80.7302231237 | 227 | 2.8e-59 |
| AMY15073.1 | MFS\_transporter | BGC0001339 | Squalestatin\_S1\_biosynthetic\_gene\_cluster | 28 | 92.4949290061 | 116 | 1.2e-25 |
| ABC87523.1 | putative\_drug\_efflux\_transporter | BGC0001011 | Meridamycin\_biosynthetic\_gene\_cluster | 30 | 28.6004056795 | 61 | 3.4e-09 |
| AAL15595.1 | Sim17 | BGC0000270 | Simocyclinone\_biosynthetic\_gene\_cluster | 30 | 34.8884381339 | 59 | 1.7e-08 |
| AAK06799.1 | putative\_export\_protein\_SimEx | BGC0001072 | Simocyclinone\_biosynthetic\_gene\_cluster | 30 | 34.8884381339 | 58 | 2.9e-08 |
